# Aqueous MXene-Assisted Charge Transport for Sliding Cu/n-Si DC Triboelectric Nanogenerators

**DOI:** 10.3390/nano16090567

**Published:** 2026-05-05

**Authors:** Dimaral Aben, Yerkezhan Amangeldinova, Dong-Myeong Shin, Yoon-Hwae Hwang

**Affiliations:** 1 Department of Nano Fusion Technology, Pusan National University, Busan 46241, Republic of Korea; 2 Crystal Bank, Pusan National University, Busan 46241, Republic of Korea; 3 Department of Mechanical Engineering, The University of Hong Kong, Hong Kong, China; 4 School of Transdisciplinary Engineering & BK FOUR Nanoconvergence Technology Division, Pusan National University, Busan 46241, Republic of Korea

**Keywords:** nanogenerator, triboelectricity, direct current, MXene, electron transport

## Abstract

This study explores the influence of MXene solution as an interfacial liquid on the output performance of a Cu/n-Si-based direct current triboelectric nanogenerator (DC-TENG) system. The ${\mathrm{Ti}}_{3}{\mathrm{AlC}}_{2}$ MAX phase was successfully transformed into ${\mathrm{Ti}}_{3}C_{2}T_{x}$ MXene through selective etching and was confirmed by scanning electron microscopy with energy-dispersive spectroscopy (SEM/EDS) and X-ray diffraction (XRD) analyses, which revealed an increase in d-spacing from 8.99 to 9.58 Å and a transition from dense layered grains to delaminated, sheet-like structures. Electrochemical impedance spectroscopy (EIS) demonstrated a pronounced reduction in impedance with the introduction of MXene solution, indicating enhanced interfacial conductivity and charge transfer capability. The presence of MXene in deionized (DI) water led to the formation of an electrical double layer (EDL) at the Cu/n-Si interface, contributing to additional interfacial capacitance and more efficient charge relaxation dynamics. As a result, the DC-TENG output was significantly enhanced with the incorporation of MXene into the system, exhibiting a markedly higher current compared to the dry contact condition. Moreover, the MXene solution helped suppress charge decay compared to dry interfaces, highlighting its role as an effective liquid medium for stabilizing surface charge and improving interfacial electron transport in DC-TENG systems.

## 1. Introduction

MXene is an emerging class of two-dimensional (2D) transition metal carbides, nitrides, and carbonitrides that has attracted attention due to its unique combination of physiochemical properties [1,2,3,4]. Typically, these materials are derived from the selective etching of the A from MAX phases and exhibit a general formula of $M_{n+1}X_{n}T_{x}$, where M represents an early transition metal, X is carbon and/or nitrogen, and T is surface termination such as -O, -OH, and -F. This structure provides MXene with properties such as electric and metallic conductivity, hydrophilicity, biocompatibility, large specific surface area, size tunability, rich surface chemistry, mechanical flexibility, and highly layered morphology.

Due to these versatile properties, MXene is considered the building block of next-generation materials and multifunctional devices. MXene and MXene-based composites have demonstrated potential in a broad range of applications, including physical and chemical sensors [5,6], electrocatalysis and photocatalysis [7,8,9], such as carbon dioxide reduction [10,11], oxygen evolution [12,13], hydrogen evolution [14,15,16], oxygen reduction [17,18], and nitrogen reduction reactions [19], energy storage systems including supercapacitors [20,21], batteries [22], and hydrogen storage devices [23], as well as biomedical applications [24]. In many of these systems, the MXene’s high conductivity and surface reactivity play an important role in facilitating charge transport and interfacial reactions.

Recently, MXene has been introduced as an active layer in triboelectric nanogenerators (TENGs) [25,26,27]. TENGs are a type of low-frequency energy harvester that can effectively collect mechanical energy from their environment and convert it into electrical energy [28,29,30,31]. They operate based on the coupling effect of contact electrification and electrostatic induction. In sliding-mode DC-TENGs, a unidirectional charge flow is achieved due to the continuous relative motion between two materials [32]. However, the performance of solid–solid interface TENGs is often limited by inefficient charge transfer and instability of the triboelectric layer [33,34,35,36]. Therefore, the identification of materials capable of enhancing charge generation, improving interfacial conductivity, and stabilizing accumulated charges remains a significant challenge.

MXene is a particularly promising material for such integration due to its excellent electrical conductivity, which facilitates rapid charge transport. Its abundant surface terminations enable strong interaction with polar liquids, and its layered architecture provides confined spaces that can accommodate ions or charges, potentially acting as charge trapping or storage sites. These features make MXene an attractive candidate for enhancing charge transfer and improving overall TENG performance, particularly in systems involving liquid–solid interactions.

In our work, we fabricated a MXene in deionized (DI) water-based DC-TENG and investigated its effect on charge enhancement. When MXene was dispersed in DI water, its layered structure interacted with the polar water molecules, forming a conductive interfacial medium at the sliding junction between the metal (Cu) and the semiconductor (n-Si). The synergistic interaction between DI water and the MXene layers promoted more efficient charge separation and interfacial charge transport. As a result, the output performance of the DC-TENG increased significantly compared to both the dry contact and the DI water-only cases. This enhancement can be attributed to strengthened interfacial polarization, the formation of continuous electrical pathways, and improved stabilization of accumulated charges within the layered MXene framework. Overall, these findings highlight the potential of MXene-based liquid systems in optimizing the performance of liquid–solid interface DC-TENGs.

## 2. Experimental Section

### 2.1. Materials

The parent MAX, ${\mathrm{Ti}}_{3}{\mathrm{AlC}}_{2}$ phase powder (99.8% trace metals basis) was used as a precursor for MXene, ${\mathrm{Ti}}_{3}C_{2}T_{x}$ synthesis and hydrofluoric acid (HF, ACS reagent 48%) as an etchant. Single-side polished n-type silicon (n-Si) wafers with a diameter of 2 inches, a crystal plane orientation of [111], a thickness of 0.5 mm, and a resistivity ranging from 100 to 3000 Ω·cm were used as a sliding triboelectric layer for device construction. All chemicals and materials were purchased from Sigma Aldrich (St. Louis, MO, USA) and used without further purification.

### 2.2. MXene Synthesis

The MXene was synthesized by etching aluminum from the parent MAX phase following the method by [2]. ${\mathrm{Ti}}_{3}{\mathrm{AlC}}_{2}$ powder was gradually added to a 30% *v*/*v* HF solution under continuous stirring. The etching reaction was carried out for 5 h at room temperature. After the etching process, the mixture underwent centrifugation, followed by repeated washing with deionized (DI) water until the pH stabilized approximately between 4 and 6. The prepared MXene slurry exhibited a dark colloidal appearance characteristic of ${\mathrm{Ti}}_{3}C_{2}T_{x}$ flakes and was stored in a refrigerator for further use.

### 2.3. Material Characterization

The surface morphology and elemental composition were characterized using scanning electron microscopy with energy-dispersive spectroscopy (SEM/EDS, JCM-7000 Neoscope, Edinburgh Instruments, Livingston, UK). The crystal structure of the samples was analyzed using powder X-ray diffraction (XRD, Xpert3, Malvern Panalytical, Almelo, The Netherlands). Electrochemical impedance spectroscopy (EIS) measurements were conducted by a µStat 400 Bipotentiostat/Galvanostat (Metrohm, Herisau, Switzerland).

### 2.4. Fabrication and Electrical Measurements of Solid–Liquid DC-TENG

The liquid–solid direct current triboelectric nanogenerator (DC-TENG) device was fabricated using copper (Cu) as both the electrode and triboelectric layer, while n-type silicon (n-Si) served as the counter sliding triboelectric layer. The MXene solution acted as the interfacial liquid medium between the Cu and n-Si surfaces. A customized well structure was designed and integrated into the device to contain the MXene solution within a defined area, ensuring stable operation during forward and backward slidings.

For electrical measurements, the device was connected to an oscilloscope (Agilent DSO-X-2014 A, Santa Clara, CA, USA) and a current preamplifier (SRS SR-570, Stanford Research Systems, Sunnyvale, CA, USA) to record output current and voltage signals. A servo motor driver (LS Mecapion APM-SB02 ADK, Daegu, Republic of Korea) was used to precisely control the linear motor’s movement that produced the sliding motion between Cu and n-Si layers. This configuration allowed us to measure the output performance of the DC-TENG under controlled conditions. The device operation was systematically evaluated under a sliding motion with a displacement of 3.0 cm, a sliding speed of 50 mm/s, an applied force of 0.5 N, and a stopping time of 3 s per cycle.

## 3. Results and Discussion

### 3.1. Structural Analysis of MAX and Synthesized MXene

**Figure 1:**
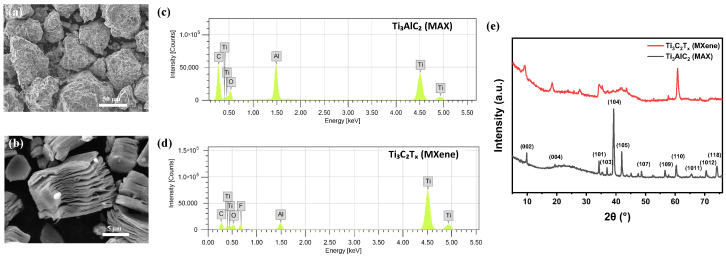
SEM images of (**a**) MAX phase and (**b**) MXene. EDS analysis of (**c**) MAX and (**d**) synthesized MXene. (**e**) XRD profiles of MAX phase and MXene.

The MXene was synthesized by selective etching of the aluminum (Al) layer from the parent ${\mathrm{Ti}}_{3}{\mathrm{AlC}}_{2}$ MAX phase using 30% *v*/*v* HF aqueous solution. The morphological characteristics of the parent MAX phase and the synthesized MXene were analyzed using SEM, as shown in Figure 1a and Figure 1b, respectively. The SEM observations demonstrated that the MXene exhibited a multilayered structure after the HF etching process, confirming the successful transformation of the MAX phase into ${\mathrm{Ti}}_{3}C_{2}T_{x}$. The ${\mathrm{Ti}}_{3}{\mathrm{AlC}}_{2}$ MAX phase showed compact, densely packed grains with layered morphology and an average particle size of less than 100 μm. After the selective removal of Al layers using hydrofluoric acid, the material evolved into a sheet-like morphology of ${\mathrm{Ti}}_{3}C_{2}T_{x}$ MXene with lateral dimensions ranging from 5 to 20 μm. The etched ${\mathrm{Ti}}_{3}C_{2}T_{x}$ displayed a delaminated, accordion-like structure composed of loosely stacked sheets, indicating the effective elimination of Al atoms and the formation of multilayered MXene with enhanced surface area and expanded interlayer spacing likely caused by hydrogen gas evolution during the etching reaction.

The energy-dispersive spectroscopy (EDS) results are presented in Figure 1c,d and Figure S1. The EDS spectrum of the MAX phase confirmed the presence of Ti, Al, and C, with a Ti to Al ratio of approximately 2:1, indicating the successful formation of ${\mathrm{Ti}}_{3}{\mathrm{AlC}}_{2}$ (Figure 1c and Table S1). After etching, the appearance of F and O elements in the MXene (Figure 1d) suggested the introduction of surface functional groups. At the same time, the significant reduction of Al content confirmed its effective removal from the MAX phase. As demonstrated in the Supplementary Table S2, the O/F ratio was found to be 1.85, and the Ti/Al ratio increased to 11.6, further verifying the elimination of Al and its substitution by surface functionalities. The selective etching process of Al leads to structural expansion and delamination, transforming the layered structure of MAX into 2D MXene nanosheets.

XRD profile of MAX phase and MXene is shown in Figure 1e. The XRD pattern confirmed the crystalline structure of the samples, with both ${\mathrm{Ti}}_{3}{\mathrm{AlC}}_{2}$ and ${\mathrm{Ti}}_{3}C_{2}T_{x}$ displaying well-defined crystalline structures. The MAX phase, ${\mathrm{Ti}}_{3}{\mathrm{AlC}}_{2}$ exhibited sharp 2θ peaks at 9.83°, 20.89°, 34.29°, 36.97°, 39.21°, 41.92°, 48.63°, 56.58°, 60.31°, 65.62°, 70.37°, and 74.10° which correspond to the (002), (004), (101), (103), (104), (105), (107), (109), (110), (1011), (1012), and (118) crystal planes, respectively [37,38]. The disappearance of the (104) peak in ${\mathrm{Ti}}_{3}C_{2}T_{x}$, MXene, indicated the breakdown of ${\mathrm{Ti}}_{3}{\mathrm{AlC}}_{2}$ and the formation of surface functional groups. A distinct 2θ peak at 9.23° corresponding to the (002) plane represented the typical feature of MXene, while additional peaks at 18.45°, 27.72°, 34.23°, 43.59°, and 60.83° verified its crystalline nature. The disappearance of the ${\mathrm{Ti}}_{3}{\mathrm{AlC}}_{2}$ peak at 39.21° and the emergence of broad peaks of ${\mathrm{Ti}}_{3}C_{2}T_{x}$ at 34.23° and 41.82° confirmed the removal of Al layers and the introduction of surface terminations. Moreover, the d-spacing increased from 8.99 Å for the MAX phase to 9.58 Å for MXene, indicating interlayer expansion caused by the etching process and the incorporation of surface functional groups.

### 3.2. Triboelectric Output of Solid–Liquid DC-TENG

**Figure 2:**
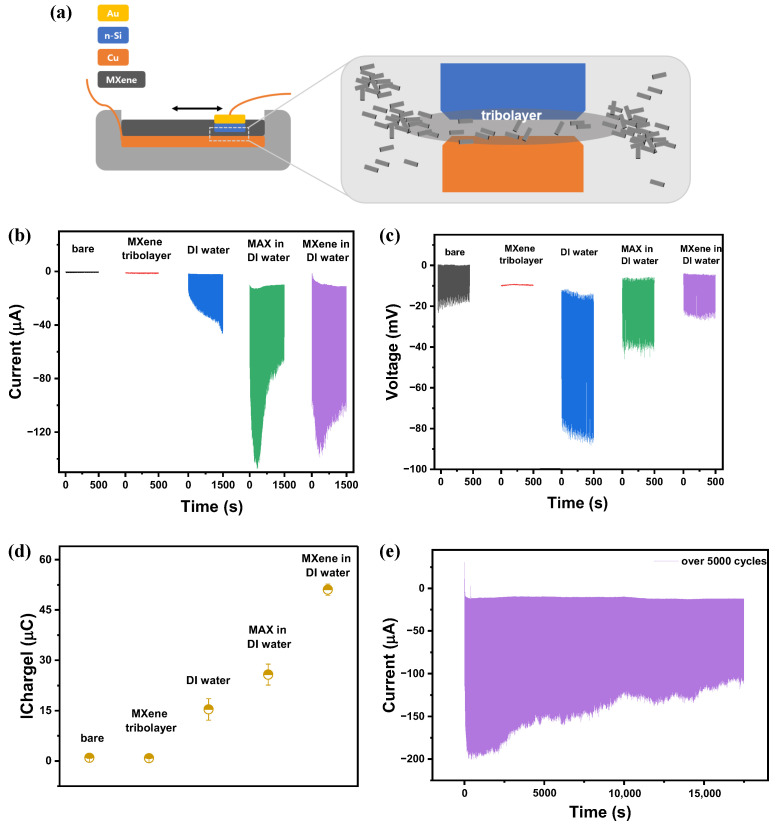
(**a**) Schematic of the sliding-mode liquid–solid DC-TENG illustrating n-Si and Cu interfacial contact and dynamic tribolayer formation during sliding. Electrical output performance under different interfacial conditions: dry contact (bare), MXene tribolayer, DI water, MAX phase, and MXene dispersed in DI water. (**b**) Output current, (**c**) output voltage, (**d**) transferred charge, and (**e**) durability performance of the MXene in DI water-based DC-TENG over 5000 sliding cycles.

In this study, we systematically investigated the effect of MXene dispersed in DI water at the metal-semiconductor interface on the output performance of a sliding-mode DC-TENG. As illustrated in Figure 2a, n-Si and Cu were used as the sliding triboelectric pair, and MXene in DI water was introduced at their interface and confined within a polymer well to maintain a controlled liquid environment. The device operated at a sliding velocity of 50 mm/s, a travel distance of 30 mm, a physical load of 0.5 N, and a stopping time of 3000 ms. A total volume of 2.5 mL solution was introduced at the interface, and the system was driven in forward and backward sliding motion. After repeated sliding in MXene aqueous solution, a MXene-containing tribolayer forms on the surface due to solvent evaporation and material transfer, altering the interfacial electrical properties. In this system, the MXene solution acts as both a lubricating interfacial layer that stabilizes the sliding contact and a charge transport medium that facilitates charge transfer in the DC-TENG.

Figure 2 presents the device configuration and electrical output characteristics of the sliding-mode liquid–solid DC-TENG, including current, voltage, transferred charge, and durability under various interfacial conditions such as dry contact, MXene tribolayer, DI water, MAX aqueous solution, and MXene dispersed in DI water. As shown in Figure 2b, the dry interface exhibited the lowest steady-state current (∼−0.43 μA). The steady-state current magnitude exhibited an increasing trend from MXene tribolayer (∼−0.47 μA), DI water (∼−46.7 μA), MAX (∼−56.0 μA), to MXene in DI water (∼−78.8 μA), with the MXene aqueous system showing the highest output. The introduction of DI water significantly enhanced the output current. Although DI water has a low ionic concentration, it contains dissolved species that facilitate electrical double layer (EDL) formation at the Cu/n-Si interface [30]. The EDL reduces effective contact resistance and promotes interfacial charge transfer, thereby resulting in higher current compared to dry contact. For MAX and MXene dispersed in DI water, the output current initially increased and then reached a stable region after prolonged sliding. This suggests that time is required for the formation and stabilization of a MAX- or MXene-induced tribolayer. The output current of MAX stabilized at approximately −56.0 μA, whereas MXene in DI water reached approximately −78.8 μA, exhibiting the highest performance. The improved output of MXene is attributed to its 2D layered structure, which provides a higher surface area and enhanced interfacial charge interaction compared to the parent MAX phase.

In terms of output voltage, shown in Figure 2c, the bare interface showed −20 mV, while the MXene tribolayer exhibited the lowest voltage of −0.7 mV. The voltage generation in dry contact is mainly governed by the built-in potential difference due to the work function asymmetry between n-Si and Cu [35]. DI water produced a higher voltage of −70 mV, likely due to improved effective contact area and enhanced interfacial charge separation at the interface between n-Si and Cu. MAX showed moderate voltage enhancement. However, DC-TENG with MXene in DI water exhibited a voltage of approximately −20 mV, showing a trend similar to that of the DC-TENG without the solution. This can be explained by the high conductivity of MXene, which provides leakage pathways for accumulated charges, leading to rapid charge dissipation and electric field screening, thereby restraining potential buildup at the interface. In addition, the formation of EDL at the liquid–solid interface can dynamically regulate charge distribution via ion transport and ion-electron coupling processes [39,40]. While such EDL-mediated effects can contribute to electrical output, in this system the dominant effect appears to be charge screening due to the high conductivity of MXene.

The induced charge values shown in Figure 2d were derived by integrating current peaks, and they followed the same trend as the output current. The values were 0.95, 0.83, 15.4, 25.8, and 51.1 μC for bare, MXene tribolayer, DI water, MAX, and MXene aqueous solutions, respectively. Normalization by the active sliding area (1cm×3cm) gave surface charge densities of 0.32, 0.28, 5.13, 8.60, and 17.03 $\mu C\;{\mathrm{cm}}^{-2}$, respectively. An overview with representative semiconductor-based liquid–solid DC-TENG systems is summarized in Table 1, enabling direct comparison with the reported values. These results confirm that a conductive and ionically active medium, such as MXene in DI water, significantly enhances interfacial charge generation.

The durability testing of MXene in DI water-based DC-TENG over 5000 cycles as shown in Figure 2e, demonstrated sustained output with gradual decay. The decrease is attributed to dynamic redistribution or partial removal of liquid and MXene species during prolonged sliding, while maintaining stable long-term operation.

**Figure 3:**
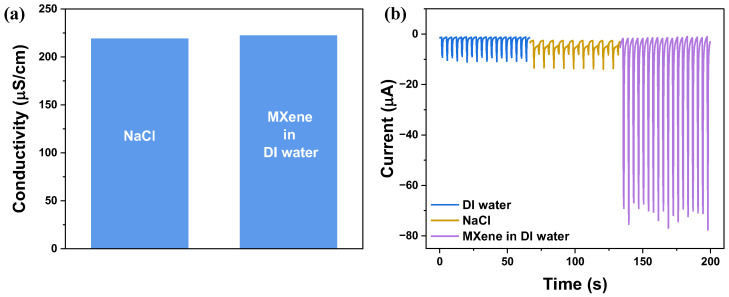
Decoupling ionic conductivity effects using conductivity-matched electrolytes. (**a**) Electrical conductivity of NaCl solution and MXene in DI water. (**b**) Output current signals measured under different liquid conditions: DI water, NaCl and aqueous MXene solutions.

To decouple the effects of ionic conductivity from the intrinsic role of MXene, control experiments were conducted using DI water, NaCl solution ($218\;\mu S\;{\mathrm{cm}}^{-1}$) with conductivity matched to that of the MXene dispersion ($222\;\mu S\;{\mathrm{cm}}^{-1}$) (Figure 3a). As shown in Figure 3b, DI water and NaCl generated comparable current outputs of −8.95 ± 0.26 and −8.17 ± 0.18 μA, respectively. This indicated that increasing ionic strength alone does not significantly enhance the device performance. In contrast, the MXene system exhibited approximately nine times greater current output (−72.5 ± 1.56 μA) than both DI water and NaCl. This demonstrated that the enhancement cannot be explained solely by ionic conductivity. Instead, MXene introduced additional interfacial contributions due to its intrinsic properties, such as surface terminations and ability to promote interfacial charge transfer, leading to improved device performance. Additional representative voltage signals are provided in Figure S2, where the NaCl and MXene in DI water cases exhibit similar voltage responses, while DI water shows a higher voltage amplitude. This observation further supports that increased ionic conductivity leads to enhanced charge screening and reduced potential buildup.

### 3.3. Electrochemical Impedance Analysis

**Figure 4:**
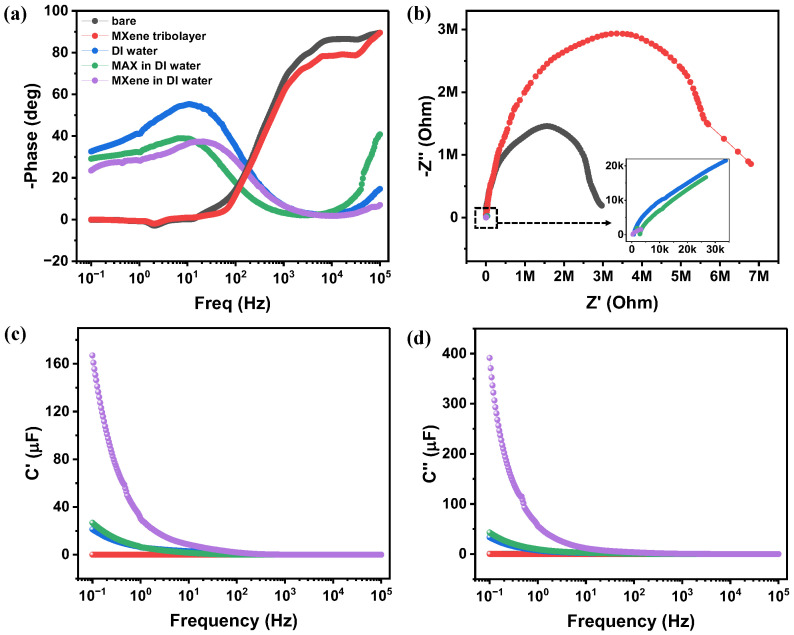
Electrochemical impedance spectroscopy (EIS) analysis of the sliding DC-TENG under different interfacial conditions: bare, MXene tribolayer, DI water, MAX phase, and MXene dispersed in DI water. (**a**) Phase angle as a function of frequency, (**b**) Nyquist plots, (**c**) real part of capacitance ($C^{\prime }$), and (**d**) imaginary part of capacitance ($C^{\prime \prime }$) as a function of frequency.

Electrochemical impedance spectroscopy (EIS) analysis further supports the electrical output results. As shown in Figure 4a, the Bode phase plot indicated that at higher frequencies, the dry surface and MXene tribolayer approach a more capacitive region (∼90° phase angle). In contrast, the liquid containing systems, such as DI water, MAX, and MXene in DI water, exhibited behavior closer to resistive conduction. At lower frequencies, typically below 1 kHz, EDL formation became more prominent since ions in the solution have sufficient time to migrate, accumulate at the surfaces, thereby forming the ionic layer. This low-frequency response indicates dominant ionic transport and interfacial polarization processes. In the dry contact configuration, the system behaved predominantly resistively, which limits charge transfer across the interface, resulting in the suppressed electrical output. A secondary relaxation peak observed at lower frequencies suggests the coexistence of both fast and slow interfacial charge relaxation processes. This low-frequency response arises from the formation of an electrical double layer (EDL) at the interface between the liquid-assisted systems and the triboelectric surfaces, contributing to the enhanced interfacial capacitance and improved charge dynamics.

The Nyquist plots demonstrated in Figure 4b further confirm these differences. A progressive reduction in impedance is observed when the system is shifting from the dry condition to the liquid-mediated systems, suggesting improved interfacial conductivity and reduced charge transfer resistance. The semicircle diameter in the high-frequency region, representing charge transfer resistance, decreased significantly in the presence of DI water, MAX, and MXene in DI water. In the dry case, the absence of a liquid medium resulted in high interfacial resistance and limited effective contact between metal and semiconductor surfaces, leading to negligible current generation.

The introduction of DI water reduced the impedance from megaohms to the kiloohms region by facilitating electrical double layer formation and strengthening interfacial coupling. This can explain the simultaneous increase in current and voltage observed in the electrical measurements. This behavior indicates a transition from predominantly electronic contact-limited transport in the dry state to ionic-assisted charge transport in the liquid-mediated systems. When conductive MXene sheets are added to the aqueous solution, the internal resistance decreased even further due to the enhanced surface conductivity and additional active sites for charge transport. As a result, the current generation improved significantly. However, the improved conductivity also accelerated charge dissipation, which suppressed the voltage buildup. This indicates that the system transitions from capacitance-dominated behavior to transport-dominated charge dynamics in the MXene-mediated configuration.

As illustrated in Figure 4c,d, the capacitance components were extracted from the impedance analysis. The real part of capacitance ($C^{\prime }$) represents the energy storage capability, while the imaginary part of capacitance ($C^{\prime \prime }$) corresponds to the energy dissipation or dielectric loss within the system. The energy storage ability of the system was enhanced with liquid medium addition from nearly negligible capacitance in dry contact and MXene tribolayer to ∼30 μF for DI water and MAX solutions, and further increasing to ∼170 μF for the MXene aqueous solution. These extracted capacitance values were used as a quantitative indicator of interfacial charge storage and transport efficiency.

Notably, in the MXene dispersed in DI water solution, the imaginary part of capacitance (∼400 μF) was found to be higher than the real capacitance, indicating increased energy dissipation compared to other conditions. This behavior can be attributed to the dynamic ion movement and polarization relaxation within a liquid environment containing MXene nanosheets. The surface terminations of MXene (-O, -OH, -F) and its high surface area facilitate rapid ion adsorption and desorption, introducing additional relaxation processes and interfacial polarization. Consequently, while the MXene–DI water interface improved charge transfer through enhanced ionic conduction, it also promoted dielectric loss due to continuous charge recombination within the interfacial layer. Therefore, the MXene mediated EDL system demonstrated a trade-off between conductivity enhancement and energy dissipation.

### 3.4. Working Mechanism of the Solid–Liquid DC-TENG

**Figure 5:**
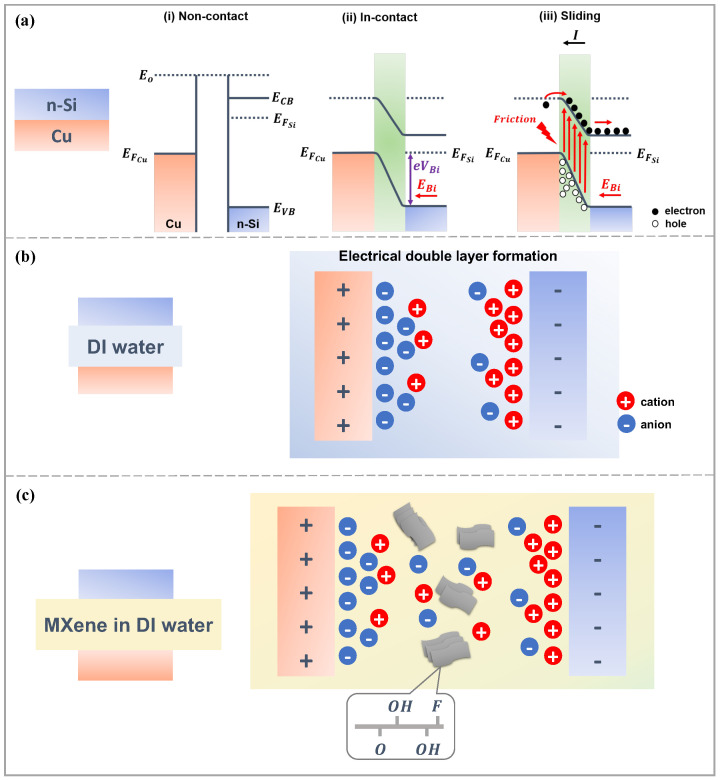
Schematic illustration of the proposed working mechanism of the MXene-assisted DC-TENG. (**a**) Energy band diagrams of the Cu/n-Si junction under (**i**) non-contact, (**ii**) contact, and (**iii**) sliding states, where the red arrows indicate the direction of electron flow and the built-in electric field during sliding. (**b**) Formation of the electrical double layer (EDL) at the Cu/DI water/n-Si interface. (**c**) MXene-assisted charge transport via conductive bridging at the Cu/MXene solution/n-Si interface.

The schematic in Figure 5 illustrates the proposed working mechanism of the n-Si/Cu DC-TENG under (a) bare contact, (b) DI water, and (c) MXene-assisted conditions. The mechanism evolves from a purely solid–solid semiconductor-metal junction (bare contact) to a liquid-mediated interface (DI water), and finally to a conductive interfacial system (MXene-assisted), resulting in progressively enhanced charge transfer. In the bare contact configuration (Figure 5a(i)), the fundamental driving force of the device originates from the work function difference between n-Si and Cu. The work functions of n-Si (4.2–4.3 eV) and Cu (∼4.7 eV) create an asymmetry that favors charge flow from n-Si to Cu during sliding motion. Initially, prior to sliding, a Schottky contact is formed at the metal–semiconductor (MS) interface, resulting in electron transfer from n-Si to Cu due to the difference in Fermi levels. This establishes a built-in electric field at the MS interface, followed by upward band bending and the formation of a thin space charge region on the n-Si side (Figure 5a(ii)). Upon the onset of sliding motion, nonequilibrium charge carriers are generated by friction and can move either along or against the built-in electric field. Under this field, electrons drift within n-Si toward the electrode, while holes move toward the MS junction, leading to electron flow through the external circuit and generation of current, as demonstrated in Figure 5a(iii). However, in the absence of an interfacial medium, charge generation and transport remain limited, resulting in relatively low current output. When the sliding motion stops, the built-in electric field is reestablished and charge flow diminishes.

When DI water is introduced (Figure 5b), the interfacial environment is modified by the formation of an electrical double layer (EDL). In the presence of DI water, polarization occurs at both n-Si and Cu interfaces, generating positively and negatively charged layers that constitute the EDL. This liquid-assisted interface enhances interfacial charge separation and increases the effective interfacial capacitance, resulting in higher voltage output compared to the bare case. However, due to the low ionic strength of DI water, charge transport remains limited, leading to only moderate current enhancement. Control experiments using conductivity-matched NaCl solution demonstrate that increasing ionic concentration alone does not significantly improve the output compared to DI water, indicating that simple ionic conduction is not the dominant factor. This suggests that bulk ionic conductivity alone does not govern the device performance.

As illustrated in Figure 5c, the introduction of MXene nanosheets further enhances this interfacial process. MXene flakes, possessing abundant surface terminations (-O, -OH, -F), act as conductive bridges that facilitate charge transfer through the liquid medium. Because DI water alone exhibits moderate ion mobility, the addition of MXene significantly reduces the interfacial impedance, thereby improving overall conductivity. Electrochemical impedance spectroscopy (EIS) confirms this by showing lower impedance values for the MXene–DI water system compared to either DI water or dry MXene alone, indicating more efficient charge transport across the interface. The MXene, synthesized in HF and primarily terminated with -F, -O, and -OH groups, exhibits a work function in the range of 3.8–5.0 eV [46,47]. This range suggests that MXene can act as an intermediate energy layer between n-Si and Cu, facilitating interfacial electron exchange. Hence, it was assumed to lie energetically between n-Si and Cu. This intermediate position allows MXene to act as an effective mediator, overcoming the Fermi level mismatch between the two electrodes and enhancing interfacial electron exchange. Overall, the MXene dispersed in DI water solution forms a stable, conductive EDL that not only reduces the charge transfer resistance but also promotes efficient coupling between triboelectric and electrochemical processes within the DC-TENG.

**Figure 6:**
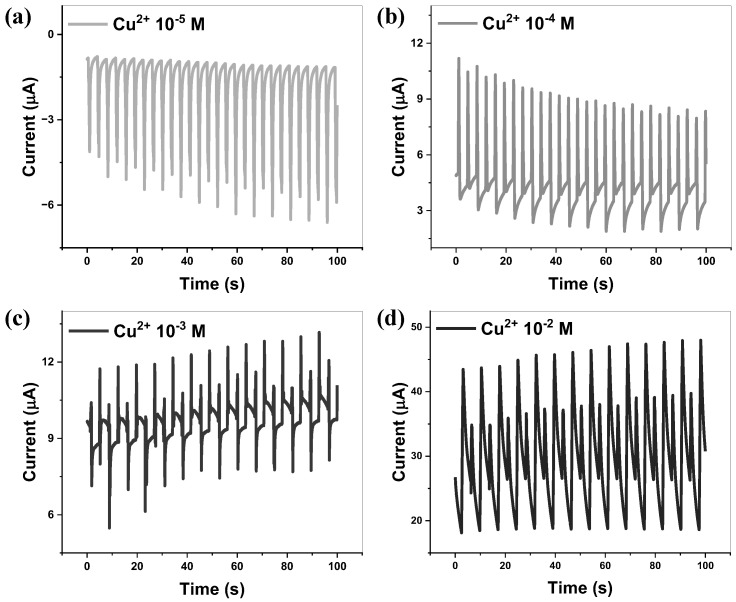
Output current of the n-Si/Cu DC-TENG measured using Cu^2+^-containing aqueous solutions with varying concentrations: (**a**) 10^−5^, (**b**) 10^−4^, (**c**) 10^−3^, and (**d**) 10^−2^ M.

To further verify that the observed behavior is not dominated by electrochemical processes associated with Cu oxidation, additional control experiments were conducted. Since the device is based on a Cu/n-Si interface, the possibility of Cu ion involvement must be considered, particularly given that Cu can undergo oxidation and contribute to EDL-related effects. To evaluate the possible contribution of Cu ion leaching to the device performance, control experiments were conducted using ${\mathrm{Cu}}^{2+}$-containing aqueous solutions with concentrations of $10^{-5}$, $10^{-4}$, $10^{-3}$, and $10^{-2}$ M, as shown in Figure 6. At the lowest concentration ($10^{-5}$ M), the current output is comparable to that obtained with DI water, indicating that trace levels of ${\mathrm{Cu}}^{2+}$ do not significantly influence the device response. As the ${\mathrm{Cu}}^{2+}$ concentration increased, the electrical conductivity of the solution correspondingly increased, as shown in Table S3. However, the output signals became unstable and exhibited distorted peak shapes. This behavior differed from the stable and enhanced current observed in the MXene-assisted system, suggesting that increased ionic concentration from ${\mathrm{Cu}}^{2+}$ does not lead to improved or consistent device performance. Furthermore, to directly evaluate the presence of Cu ions after operation, the working solution was analyzed using 5 M ammonia solution, which forms a characteristic deep blue complex in the presence of ${\mathrm{Cu}}^{2+}$ ions. As shown in Supplementary Figure S3, no visible color change was observed even after extended sliding durations (up to 180 min), indicating that Cu ion leaching, if present, is below the detectable limit under the present experimental conditions. These results indicate that the observed device performance cannot be attributed to Cu ion-induced effects, and that electrochemical contributions from Cu oxidation are minimal. Instead, the behavior is governed primarily by interfacial charge transfer processes.

In contrast to DI water and conductivity-matched electrolyte systems, the MXene-assisted configuration exhibits a substantially higher current output, indicating that its role extends beyond simple ionic or EDL effects. Therefore, the overall current generation mechanism can be described as a coupled and hierarchical process: the work function difference establishes the driving force for charge transfer (bare contact), the liquid introduces EDL-assisted interfacial charge separation (DI water), and MXene further amplifies the process by enabling efficient interfacial charge transfer and transport.

### 3.5. Effect of Wettability and Contact Conditions on Output Performance

To further understand the origin of the current generation in MXene aqueous solution-assisted DC-TENG, the relationship between MXene concentration and wettability at the solid–liquid interface was investigated. After chemical etching in hydrofluoric acid, the multilayered MXene becomes hydrophilic and carries a negative surface charge due to surface terminal groups (-O, -OH, -F). These functional groups can form hydrogen bonds with water molecules, while the high negative surface charge helps to stabilize the dispersion through electrostatic repulsion, thereby enabling the formation of a stable colloidal solution without the need for surfactants. As the MXene concentration in the colloidal solution increases, the amount of hydrophilic functional groups and MXene flakes in the liquid increases as well, which alters the interfacial energy and interaction between the liquid and the solid surface. As a result, this influences the liquid spreading behavior where one can see an improved wettability and a decrease in contact angle of the affected surface. Therefore, the wettability can serve as an indirect indicator of the MXene concentration in the solution.

**Figure 7:**
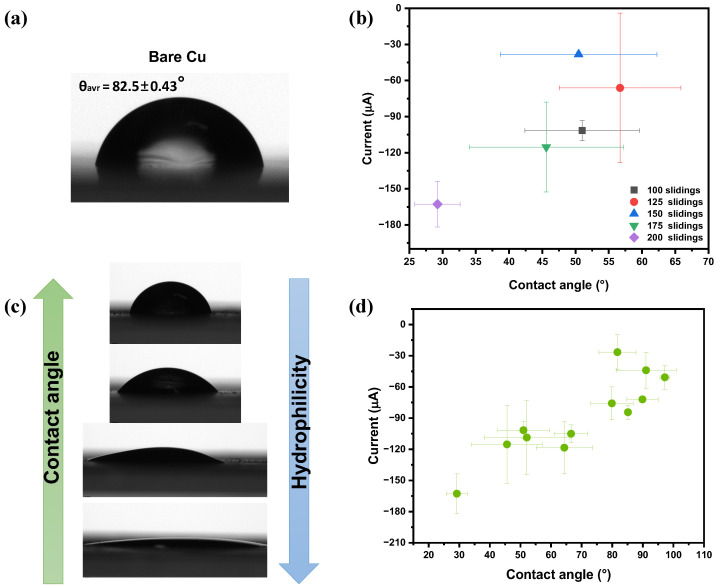
(**a**) Contact angle images of Cu surface after continuous sliding showing hydrophilicity change due to MXene tribolayer formation. (**b**) Output current as a function of contact angle after 100, 125, 150, 175, and 200 sliding cycles. (**c**) Cu surface after continuous sliding illustrating the transition from hydrophobic to hydrophilic due to MXene tribolayer formation. (**d**) Output current as a function of contact angle.

As shown in Figure 7, the wettability of MXene-mediated surfaces was analyzed after device operation by drying the surfaces overnight. The contact angle of the bare Cu surface before exposure to any solution was 82.5 ± 0.43° (Figure 7a). After contact with the MXene solution, the surface wettability varied depending on the quality and distribution of the formed tribolayer. Since the sliding is a dynamic process, the MXene particles dispersed in DI water also move continuously during operation. As a result, the exposed MXene tribolayer at the n-Si/Cu interface changes from measurement to measurement, leading to variations in the local surface condition.

As illustrated in Figure 7b, no clear dependence on the number of sliding cycles was observed, suggesting that the output performance is governed by the local distribution and quality of the MXene tribolayer rather than sliding count. Multiple contact angle measurements were taken at different positions to account for this non-uniformity and resulting variation is shown by the error bars in Figure 7d.

The contact angle images illustrating the transition from hydrophobic to hydrophilic surfaces are shown in Figure 7c. It was observed that more hydrophobic surfaces with contact angles around 100° resulted in lower current output of approximately −30 μA. In contrast, when the surface became more hydrophilic, with contact angles ranging from 50 to 30°, the current output increased significantly from −120 to −160 μA. This indicates that the current generation in MXene-assisted DC-TENG is dependent on surface wettability.

The improved performance on hydrophilic surfaces can be attributed to enhanced liquid spreading and a larger effective interfacial area between solid and liquid, which facilitates more uniform electrical double layer formation. A well-spread MXene tribolayer promotes interfacial coupling and provides continuous ionic pathways, enabling charge transfer. While poor spreading on more hydrophobic surfaces reduces the effective contact area and limits interfacial interaction, thereby hindering charge transport and decreasing the current generation.

## 4. Conclusions

In this study, we found that the incorporation of MXene solution as an interfacial medium significantly enhanced the output performance of the Cu/n-Si DC-TENG. The successful conversion of ${\mathrm{Ti}}_{3}{\mathrm{AlC}}_{2}$ MAX to ${\mathrm{Ti}}_{3}C_{2}T_{x}$ MXene was verified by XRD and SEM, showing increased interlayer spacing and the development of a sheet-like morphology. Electrochemical impedance spectroscopy confirmed that the MXene solutions effectively lowered interfacial impedance, suggesting improved conductivity and charge transfer efficiency. The formation of an electric double layer (EDL) in the MXene–DI water system introduced additional interfacial capacitance and facilitated faster charge relaxation. Overall, the MXene solution acted as a conductive and stabilizing liquid medium that enhanced interfacial electron transport, demonstrating its potential for optimizing liquid-assisted DC-TENG configurations. 

## Figures and Tables

**Table 1:** Performance comparison of semiconductor-based liquid–solid DC-TENG systems.

Material System	|Current| (μA)	|Voltage| (mV)	Liquid Volume	Contact Area	Operation Mode	Ref.
n-Si (water)	0.3	200	∼5 μL	∼0.05 cm^2^	Droplet Sliding	[41]
p-Si/n-Si (water)	0.64	300	150 μL	–	Droplet Sliding	[42]
Graphene-n-Si	2.2	300	30 μL	–	Droplet Sliding	[43]
MoS_2_	1.11	200	∼25 μL	∼1 cm^2^	Droplet Sliding	[44]
WS_2_-p-Si (NaCl)	4.2	50	2 mL	–	Lateral Sliding	[45]
MXene-n-Si/Cu	78.8	20	2.5 mL	3 cm^2^	Lateral Sliding	This work

## Data Availability

The original contributions presented in this study are included in the article/Supplementary Material. Further inquiries can be directed to the corresponding author.

## Supplementary Materials

The following supporting information can be downloaded at https://www.mdpi.com/article/10.3390/nano16090567/s1; Figure S1: SEM images of (a) MAX phase and (c) synthesized MXene. EDS analysis of (b) MAX and (d) MXene; Figure S2: Output voltage signals under different liquid conditions: DI water, NaCl, and MXene in DI water; Figure S3: The digital images showing Cu^2+^ detection in DI water using 5 M ammonia solution after DC-TENG sliding for varying durations (5 to 180 min); Table S1: Elemental analysis of parent MAX phase; Table S2: Elemental analysis of synthesized MXene; Table S3: The electrical conductivity of DI water and Cu^2+^-containing aqueous solutions with concentrations of $10^{-5}$, $10^{-4}$, $10^{-3}$, and $10^{-2}$ M.
