# Supplementary material for: Aqueous MXene-Assisted Charge Transport for Sliding Cu/n-Si DC Triboelectric Nanogenerators
Source: Nanomaterials (Basel). 2026 May 5;16(9):567. doi: 10.3390/nano16090567 (PMC13165339; doi:10.3390/nano16090567)
Supplement: Supplementary file 1 [file nanomaterials-16-00567-s001.zip › nanomaterials-4257181-supplementary.pdf]

Supplementary Materials for:

Aqueous MXene Assisted Charge Transport for Sliding Cu/n-Si DC Triboelectric Nanogenerators

Dimaral Aben[1], Yerkezhan Amangeldinova[2], Dong-Myeong Shin[3], and Yoon-Hwae Hwang[1, 4]*

[1]Department of Nano Fusion Technology, Pusan National University, Busan 46241, Republic of Korea

[2]Crystal Bank, Pusan National University, Busan 46241, Republic of Korea

[3]Department of Mechanical Engineering, The University of Hong Kong, Hong Kong, P. R. China

[4]School of Transdisciplinary Engineering & BK FOUR Nanoconvergence Technology Division, Pusan National University, Busan 46241, Republic of Korea

*Correspondence: yhwang@pusan.ac.kr

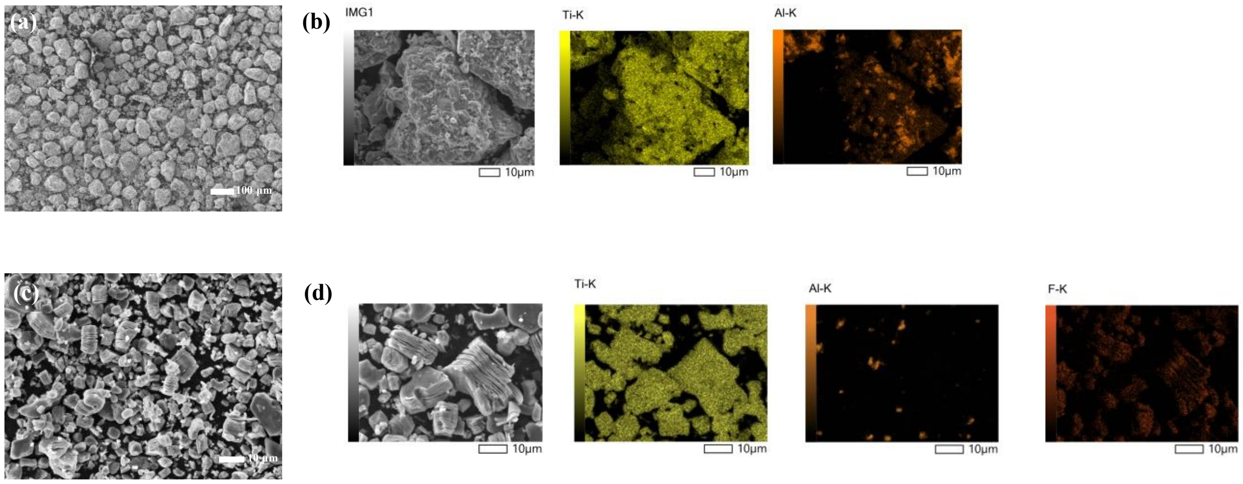

Figure S1. SEM images of (a) MAX phase and (c) synthesized MXene. EDS analysis of (b) MAX and (d) MXene

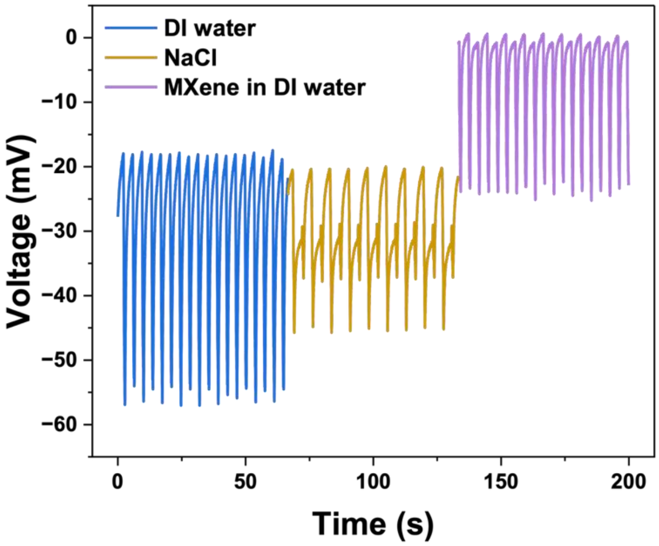

Figure S2. Output voltage signals under different liquid conditions: DI water, NaCl, and MXene in DI water

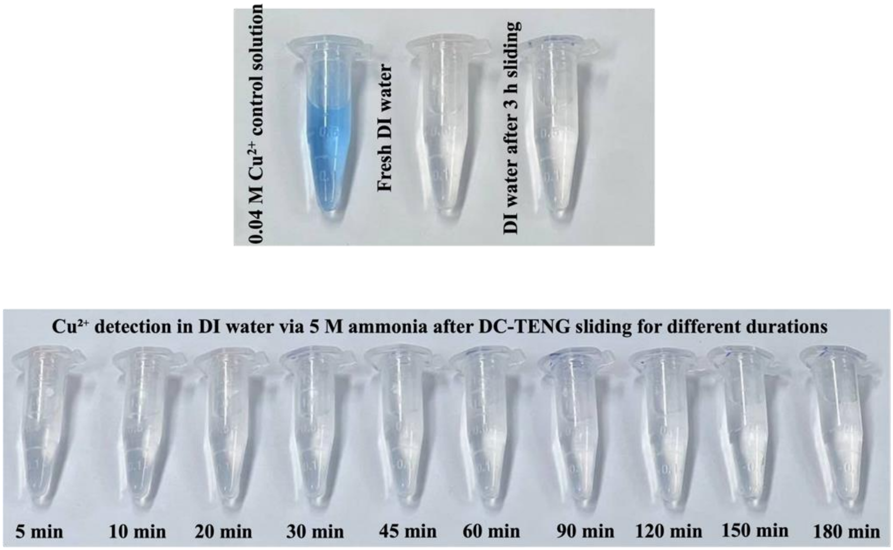

Figure S3. The digital images showing Cu2+ detection in DI water using 5 M ammonia solution after DC-TENG sliding for varying durations (5 to 180 min)

Table S1. Elemental analysis of parent MAX phase

**Elements**	**Atomic %**
C	37.08
Al	21.67
Ti	41.25
Total	100.00

Table S2. Elemental analysis of synthesized MXene

Elements	Atomic %
C	33.54
O	26.19
F	14.12
Al	2.08
Ti	24.07
Total	100.00

Table S3. The electrical conductivity of DI water and Cu^{2+}-containing aqueous solutions with concentrations of 10^{-5}, 10^{-4}, 10^{-3}, and 10^{-2} M

**Sample**	**Conductivity (μS/cm)**
DI water	1.1 ± 0.1
Cu^{2+} 10^{-5} M	12.3 ± 0.1
Cu^{2+} 10^{-4} M	115.6 ± 0.9
Cu^{2+} 10^{-3} M	1007 ± 5
Cu^{2+} 10^{-2} M	7000 ± 50
